# A 10 m resolution urban green space map for major Latin American cities from Sentinel-2 remote sensing images and OpenStreetMap

**DOI:** 10.1038/s41597-022-01701-y

**Published:** 2022-09-24

**Authors:** Yang Ju, Iryna Dronova, Xavier Delclòs-Alió

**Affiliations:** 1 School of Architecture and Urban Planning, Nanjing University, Nanjing, China; 2 Department of Environmental Science, Policy, and Management, University of California, Berkeley, USA; 3 Department of Landscape Architecture and Environmental Planning, University of California, Berkeley, USA; 4 Department of Geography, Universitat Rovira i Virgili, Vila-seca, Spain

**Keywords:** Geography, Forestry, Sustainability, Developing world

## Abstract

Mapping is fundamental to studies on urban green space (UGS). Despite a growing archive of land cover maps (where UGS is included) at global and regional scales, mapping efforts dedicated to UGS are still limited. As UGS is often a part of the heterogenous urban landscape, low-resolution land cover maps from remote sensing images tend to confuse UGS with other land covers. Here we produced the first 10 m resolution UGS map for the main urban clusters across 371 major Latin American cities as of 2017. Our approach applied a supervised classification of Sentinel-2 satellite images and UGS samples derived from OpenStreetMap (OSM). The overall accuracy of this UGS map in 11 randomly selected cities was 0.87. We further improved mapping quality through a visual inspection and additional quality control of the samples. The resulting UGS map enables studies to measure area, spatial configuration, and human exposures to UGS, facilitating studies on the relationship between UGS and human exposures to environmental hazards, public health outcomes, urban ecology, and urban planning.

## Abstract (summary)

**Table:** 

Measurement(s)	urban green space
Technology Type(s)	remote sensing • volunteered geographic information • supervised machine learning
Sample Characteristic - Environment	city
Sample Characteristic - Location	Argentina • Brazil • Chile • Colombia • Costa Rica • El Salvador • Guatemala • Mexico • Nicaragua • Panama • Peru

## Background & Summary

Urban green space (UGS) provides the residents with various ecosystem services, such as climate regulation, buffering environmental hazards, and improving physical and psychological health, making itself a vital environmental good^1–6^. Neighbourhoods lacking or with poorly maintained UGS could have elevated crime risks and adverse health outcomes such as allergies and vector-borne diseases^2,7^. Furthermore, environmental justice^8^ and green gentrification^9,10^ are inherent social challenges linked with UGS. Fine-resolution, high-accuracy UGS maps are fundamental to study the topics above in the fields of environmental health, urban ecology, and urban planning, which inform data-driven and evidence-based planning, designing, and management for UGS.

Studies have operationalized UGS as either greenness or green space maps. Greenness is calculated with spectral indices such as Normalized Vegetation Difference Index (NDVI)^11^, which are based on solar-induced reflectance of landscape features captured by air- and space-borne remote sensors. Studies show that greenness correlates with but does not directly measure biophysical properties of UGS including tree canopy cover, height, biomass, and vegetation health^11,12^. In heterogeneous urban areas, greenness of the minimum mapping units (i.e., image pixels) can be confounded by background reflectance from non-vegetated elements such as impervious surfaces^13^. Furthermore, greenness does not reflect the spatial configuration of UGS, including shape complexity (e.g, the ratio of UGS patch perimeter to area, where patch is a cluster of spatially connected UGS), patch density (i.e. number of patches over area), and connectivity (i.e. functional connections or spatial adjacency between patches), which are drawing increased attentions from research and practice^14,15^. Therefore, research findings based on greenness alone may be insufficient for providing practical guidance.

An alternative but more challenging approach uses spatially explicit UGS maps. These maps characterize physical properties of UGS including coverage and spatial patterns, which could lead to more actionable findings - for example, how the presence of large UGS patches affects the incidence of residents reporting poor health^16^. UGS maps are usually generated by classifying remote sensing images, a process requiring a substantial amount of training and validation data, remote sensing images, and computing power. Therefore, until recent advances in computing capabilities and remote sensing image archives, UGS maps over large geographic regions covering multiple cities have been relatively scarce.

The growing archive of remote sensing images from MODIS-, Landsat- and Sentinel-series, and the emergence of cloud-based image processing platforms like Google Earth Engine^17^, potentially expand the availability of UGS maps via land cover mapping efforts at global and regional scales^18^. However, data quality of such global and regional land cover maps can be unsatisfactory for studying UGS, for two main reasons. First, urbanized areas are complex landscape mosaics of UGS, built-up areas, water bodies, and undeveloped areas. Aiming at mapping the entire landscape where cities constitute only a small proportion (roughly 1–3% of the global land area in circa 2010, depending on the definition of urban land^19^), global and regional land cover maps tend to confuse UGS with built-up areas in cities. Mapping efforts tailored to cities outperform global land cover maps in mapping UGS, as illustrated by a recent study^20^. Second, major image sources for land cover mapping, such as Landsat and MODIS, are at low-to-moderate spatial resolutions (≥30 m), which are insufficient for capturing finer-scale UGS features such as street trees. Sentinel-2 satellite-based 10 m resolution land cover maps such as ESA WorldCover^21^ and FROM-GLC10^18^, which became available after the mission’s launches in 2015 and 2017, improve the mapping of fine scale UGS.

Novel data sources for UGS mapping include street-view images^22^ and LiDAR 3D scans^23^. While these data enable UGS mapping at new dimensions with high accuracy, they tend to have limited spatial and temporal coverage which may also be biased towards more affluent areas due to the high cost in data collection^24^. Therefore, the application of these novel data in research over large geographies and multiple cities is still nascent.

While UGS has been mapped in a few countries (e.g. USA and China), continents (e.g. Europe and Asia), and in all mid-and large-sized cities globally (>500,000 in population)^20^, these data remain relatively sparse in Latin America, particularly for smaller cities with population between 100,000 and 500,000. This does not align with the need for UGS maps in Latin American cities, given their high urbanization rates, fragmented built-up areas due to unplanned development, sharp socioeconomic disparities, complex urban health challenges^25,26^, and a UGS pattern different from high income countries where most UGS-related research has been conducted^27^. The objective of this study is to develop a 10 m resolution UGS map for 371 cities with over 100,000 population in 11 Latin American countries.

## Methods

### Overview

**Fig. 1:**
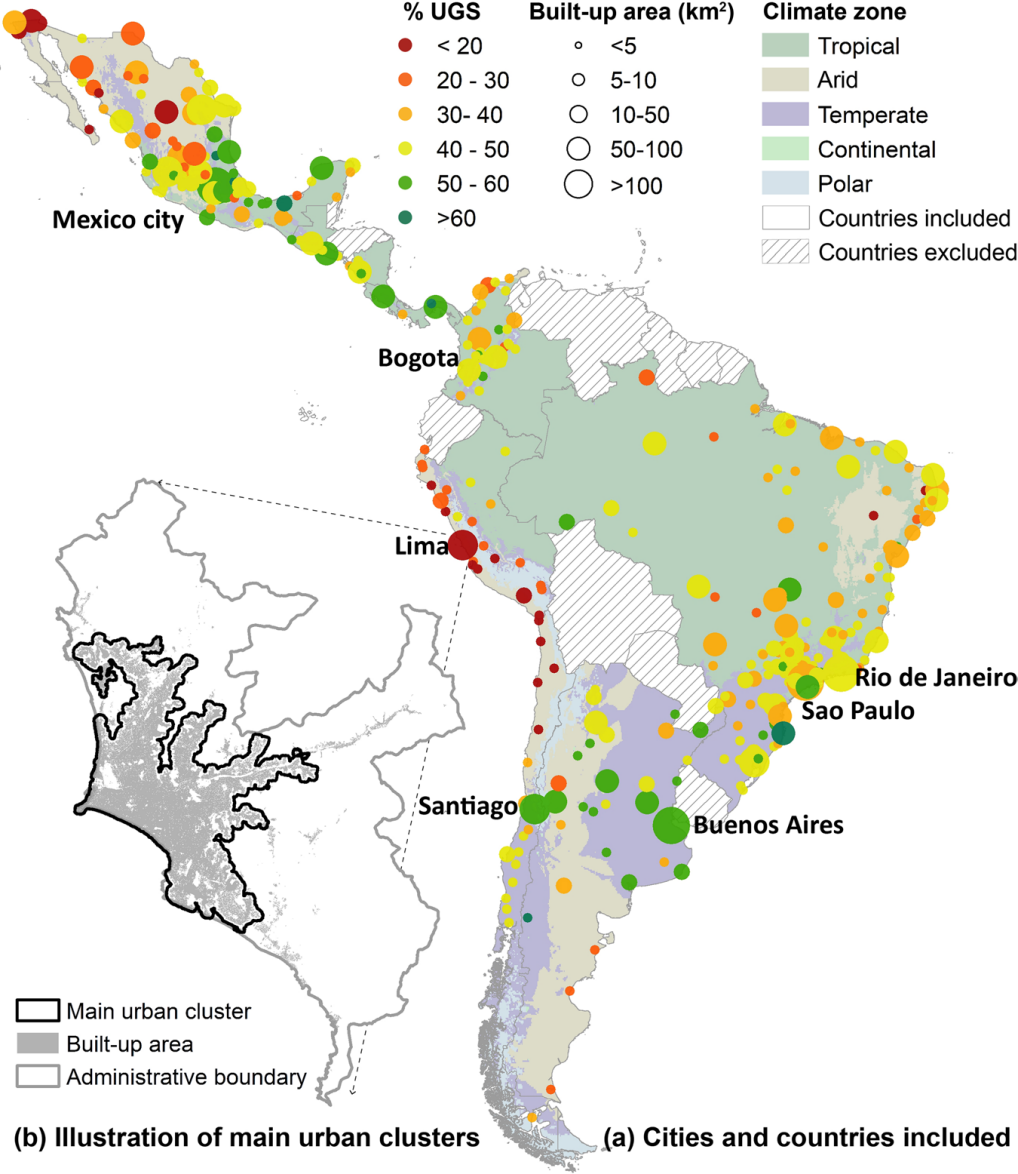
Study area (**a**) and illustration of the main urban clusters (**b**). In (**a**), markers of the cities are scaled by area of the main urban cluster and coloured by % area that is urban green space. Countries included are overlaid with their climate zones based on Koppen climate classification^42^. Inclusion and exclusion of the countries are based on the SALURBAL project^26^. In (**b**), an illustrative example of Lima shows that the majority of the city’s built-up area from Global Urban Footprint dataset^43^ is within the main urban cluster outlined by the SALURBAL project^26^.

**Fig. 2:**
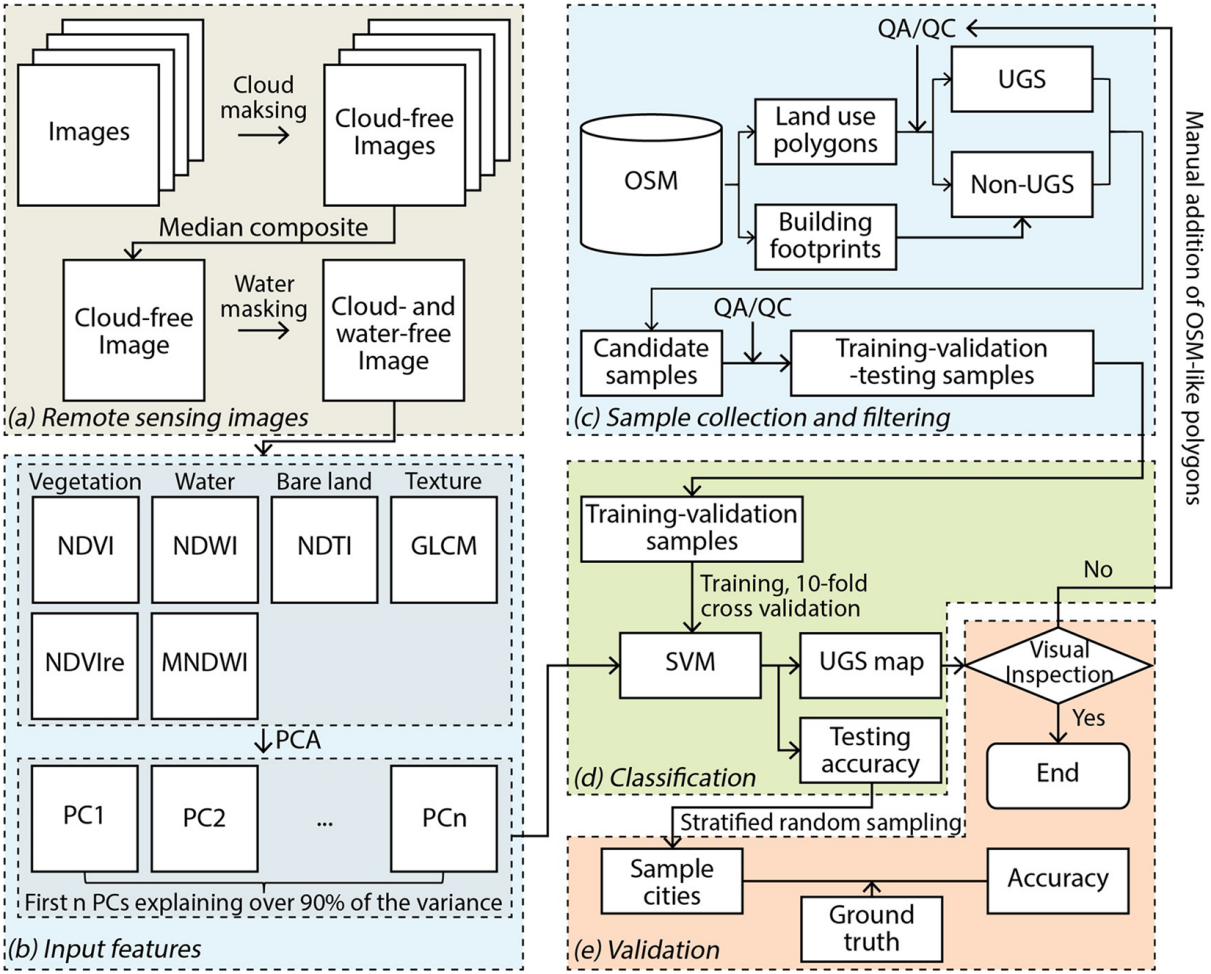
Workflow for developing the UGS map. NDVI: Normalized Difference Vegetation Index; NDVIre: red-edge-based NDVI; NDWI: Normalize Difference Water Index; MNDWI: modified NDWI; NDTI: Normalized Difference Tillage Index; GLCM: Gray Level Co-occurrence Matrix; PCA: principal component analysis; PC: principal component. OSM: OpenStreetMap; QA/QC: quality assessment and quality control; SVM: support vector machine.

To develop the UGS maps, we defined that UGS included forest, grass, shrub, agriculture, and wetland, and non-UGS included buildings, pavement, roads, barren land, and dry/non-photosynthetic vegetation. We considered that dry/non-photosynthetic vegetation as non-UGS to reduce mapping errors, because such vegetation was likely confused with barren land and some impervious surface in remote sensing images^28^. The 371 cities were identified by the Salud Urbana en América Latina (SALURBAL) study on urban health^26^, which had at least 100,000 residents in 2010 and were located in 11 countries, including Argentina, Brazil, Chile, Colombia, Costa Rica, El Salvador, Guatemala, Mexico, Nicaragua, Panama, and Peru (Fig. 1a). We focused on the main urban cluster in each city (Fig. 1b)^26^, where the landscape is highly heterogenous and dedicated UGS mapping is lacking. To perform a supervised classification of UGS from remote sensing data, we derived our training samples from a volunteer geographic information dataset, OpenStreetMap (OSM), and we used them to train a support vector machine (SVM) classifier on a cloud-free composite of all Sentinel-2 remote sensing images in 2017. To account for the inter-city differences in the biome and urban form, we performed this classification separately for each city. Below we describe the development and characteristics of this UGS map (Fig. 2).

### Remote sensing images

We used a single, median value composite of Sentinel-2 Top of Atmosphere (TOA) reflectance images in 2017^29^ as the input for the classification (Fig. 2a). Sentinel-2 images have a spatial resolution of 10 m and a temporal resolution of 5 days. For each city, we acquired and processed all images available in 2017 in Google Earth Engine, a cloud-based remote sensing image processing platform^17^.

Prior to mapping UGS, we removed cloud and water from the images using the following procedure. We first used the internal cloud mask of individual images to remove dense and cirrus cloud^30^, and we made a single median composite of these images to further remove residual clouds and to fill any gaps from the initial cloud masking. For each image pixel, the median composite takes the median of pixel values from all available images, which helped to remove cloud that had high pixel values and shadow that had low pixel values^31^. From the median composite, we masked out seasonal and permanent water bodies using a 2015 global surface water map^32^, assuming no significant changes between 2015 and 2017. The product here was a single, wall-to-wall, cloud-, shadow-, and water-free image for the year of 2017.

As a final step, we applied the boundary of the main urban cluster, as defined by the SALURBAL project^26^ to mask the remote sensing images above, so that the green space in rural areas were excluded from our UGS map. The main urban cluster is the largest continuous urban area within a city.

### Input features

We used several spectral indices (Table 1) to distinguish between UGS, impervious area, barren land, and water bodies that were the main land covers in a city (Fig. 2b)^33^. In addition, we included Gray Level Co-occurrence Matrix (GLCM) of the near infrared band to highlight the texture (i.e., spatial pattern of spectral values) of the landscape, which is expected to differ between different types of urban land cover. For example, an earlier review suggests that adding texture to the input features improves the accuracy of SVM to be comparable with more sophisticated deep neural network classifiers^34^.

**Table 1:** Input features for the classification.

Land covers	Input features	Formula
Vegetation	Normalized Difference Vegetation Index (NDVI), red-edge-based NDVI (NDVIre)	$NDVI=\frac{NIR-Red}{NIR+Red}$
		$$NDVIre=\frac{RedEdge-Red}{RedEdge+Red}$$
Water	Normalize Difference Water Index (NDWI), modified NDWI (MNDWI)	$NDTI=\frac{Green-NIR}{Green+NIR}$
		$$MNDTI=\frac{Green-SWIR1}{Green+SWIR1}$$
Impervious surface and bare land	Normalized Difference Tillage Index (NDTI)	$NDTI=\frac{SWIR1-SWIR2}{SWIR1+SWIR2}$
Texture of the landscape	Gray Level Co-occurrence Matrix of NIR	

Green: green band (wavelength: 543–578 nm); Red: red band (wavelength: 650–680 nm); RedEdge (wavelength: 698–713 nm); NIR: near infra-red band (wavelength: 785–900 nm), SWIR1: short wave infrared 1 (wavelength: 1565–1655 nm), SWIR2: short wave infrared 2 (wavelength: 2100–2280 nm).

We performed a Principal Component Analysis (PCA) to the spectral indices and GLCM (Table 1) to reduce their dimensionality and to accelerate model training. Due to multicollinearity in the spectral indices and GLCM, adding additional indices may only provide marginal gain in classification accuracy while extending model training time, limiting the efficiency to map UGS at scale. Therefore, we transformed the spectral indices and GLCM to a set of principial components (PCs) that were independent of each other. Higher order PCs, ranked by the percent variance in the untransformed data explained, tend to enhance the contrast between predominant landscape features. We selected for each city the first several PCs together explaining over 90% of the variance to reduce the dimensionality of input features.

### Sample collection and filtering

We used land use and building polygons in OSM as of Jan 30^th^, 2019 as the pool to generate training-validation-testing samples for the classification (Fig. 2c). As a volunteered geographic information dataset, OSM products vary in the availability and quality among cities, and smaller cities tended to have less coverage and lower quality as we observed. Given this limitation, we applied the following quality controls to the polygons. First, according to our earlier definition of UGS (forest, grass, shrub, agriculture, wetland) and non-UGS (buildings, pavement, roads, barren land, and dry vegetation), we divided the land use polygons into UGS and non-UGS according to their labels in OSM (Table 2). A few items are worth highlighting here. First, OSM uses a two-level labelling system (OSM key- OSM value), and we classified the land use polygons based OSM value which was indicative of detailed land use. Second, we added an additional type of mixed UGS to our earlier definition of UGS to incorporate the land use polygons that likely contained both UGS and non-UGS. Third, we acknowledged that the UGS land use polygons might still contain non-UGS such as pavement and artificial grass, so we filtered the samples derived using their NDVI values as described later. Lastly, any OSM values not listed in Table 2 were considered as non-UGS, but we omitted them for display purpose.

**Table 2:** OSM land use polygon features considered as UGS.

UGS type	OSM Key	OSM Value
Forest	Land use*	forest, wood
Grass	Land use*	grass, meadow,
	Leisure	miniature golf, sports center
	Natural	grassland
	Sport	American football, bowls, Canadian football, cricket, croquet, dog racing, equestrian, golf, horse racing, lacrosse model aerodrome, obstacle course, rugby league, rugby union, soccer
Shrub	Natural	health, scrub
Agriculture	Historic	farm
	Land use*	allotments, farmland, orchard, plant nursery, vineyard
Wetland	Natural	wetland
Mixed	Land use*	recreation ground, village green
	Leisure	disc golf course, garden, nature reserve, park
	Natural	fell
	Sport	orienteering
	Tourism	camp site

*“Land use” is a key used in OSM. However, in this study all the other keys in the table are considered as land use according to its common definition. Any OSM values not listed in here are considered as non-UGS but are omitted from the table for display purpose.

Next, we removed outliers in land use polygons using their size and shape, assuming that these outliers were delineated at low quality and therefore had incorrect labels or contained multiple land uses, which were not suitable for generating ‘pure’ samples (i.e., contained only their respective land covers) for the classification. Specifically, we retained land use polygons that were: (1) within the middle 95% of polygon area distribution, (2) in the lower 90% of polygon shape index distribution, where higher values indicated greater shape complexity. We determined these thresholds empirically, therefore a sensitivity analysis would be beneficial for future research exploring the utility of OSM in land cover mapping. Finally, we ingested the building polygons without any modification and labelled them as non-UGS, assuming that buildings were delineated and labelled with higher precision. The combined UGS and non-UGS polygons after this quality control process served as the pool for sample generation.

We generated candidate samples from the polygons discussed above and further removed outliers in these samples as follows. We conducted a regular sampling with 10 m spacing (i.e. the spatial resolution of Sentinel-2 images) within the polygons from previous steps. Since a polygon may still contain more than one land cover, we filtered the candidate samples based on their NDVI and PC values in two steps. First, since high NDVI indicated a higher likelihood of being UGS, we removed UGS samples with NDVI less than 0.1 and non-UGS samples with NDVI greater than the median NDVI of the filtered UGS samples. Second, we applied the local outlier factor (LOF)^35^ to the samples from the first step to exclude those with outlying PC values. LOF also calculated a weight indicating the likelihood of a sample being an inlier, which we used later in training the classifier.

After the filtering above, for each city we generated a training-validation-testing set for the supervised classifier by randomly selecting the candidate samples. We randomly selected 20% of the UGS and the non-UGS candidate samples respectively into an initial training-validation-testing set, and we balanced this set so that UGS and non-UGS sample sizes (i.e. number of samples) were equal. Next, we restricted the size of this set to be between 400 and 5000 samples for each city to prevent underfitting or extended training time of the classifier. It was possible that in some cities the initial training-validation-testing set exceeded or fell short of these limits. When the initial training-validation-testing set was more than 5000 samples, we instead randomly selected 2500 samples for UGS and non-UGS respectively from the candidate samples. When the initial training-validation-testing set was less than 400 samples, we used the entire pool of candidate samples and balanced the UGS and non-UGS samples. We used 75% of the training-validation-testing set for training and validation and the remaining 25% for testing.

### Supervised classification with support vector machine

We performed a supervised classification of the remote sensing images with the support vector machine (SVM) classifier^36^ to map UGS (Fig. 2d). Literature indicates that SVM outperforms other non-deep machine learning classifiers, such as decision tree, random forest, and maximum likelihood, in land cover classification^34^. In addition, when using texture-related features such as GLCM in our study, the performance of SVM is comparable with deep neural network classifiers^34^.

Our SVM classifier used a radial basis function kernel, and we tuned the model for two parameters, which were the regularization parameter (C) and kernel coefficient (gamma), following the recommendations by Hsu *et al*.^37^ For each parameter, we included 16 candidate values in an exponential sequence (2^*n*^, *n* = −3, −2, …, 12). We tuned the model using a grid search method, which iteratively trained and validated the model using each unique combination of the two parameters’ candidate values. In each iteration, we did a 10-fold cross validation using the training-validation set and measured average model performance. The set of parameter values producing the highest F1-score, which is the harmonic mean of precision and recall (i.e., measures reflecting under- and over-mapping), was used to specify the final model. We applied the final model to the testing set and recorded its F1-score as the final model accuracy, which we used later in technical validation as a stratum to sample cities for an additional accuracy assessment with independently collected samples (Fig. 2e).

### Limitations and future research directions

Several limitations and future research directions in developing UGS map are worth highlighting here. First, the mapping outcome does not distinguish between specific types of UGS in terms of their land cover/use, such as forest, grass, agriculture, park, and nature reserves. In addition, we did not include in the UGS dry or non-photosynthetic vegetation, due to its similarities to barren land and some impervious surfaces in remote sensing images^28^. Targeted mapping efforts and novel methods oriented towards detailed UGS types may help further resolve this limitation. For example, while we used an annual summary of remote sensing images (i.e. a single, median value composite of all images from 2017) that were time-invariant to map UGS, studies may use a time-series of images and extract phenological information to enable more detailed mapping of vegetation types^38^.

Second, several decisions employed in this mapping, such as those to filter OSM land use polygon using shape complexity and outlier removal based on NDVI, have yet to be tested with comparative studies to understand their implications in mapping quality. Lastly, we found that the availability, and quality to a less extent, of OSM polygons tended to be lower in small cities, prohibiting a fully automated mapping process. In these cases, manual addition and editing of OSM(-like) polygon may become necessary to ensure mapping quality (described later in Visual inspection and quality control).

## Data Records

**Fig. 3:**
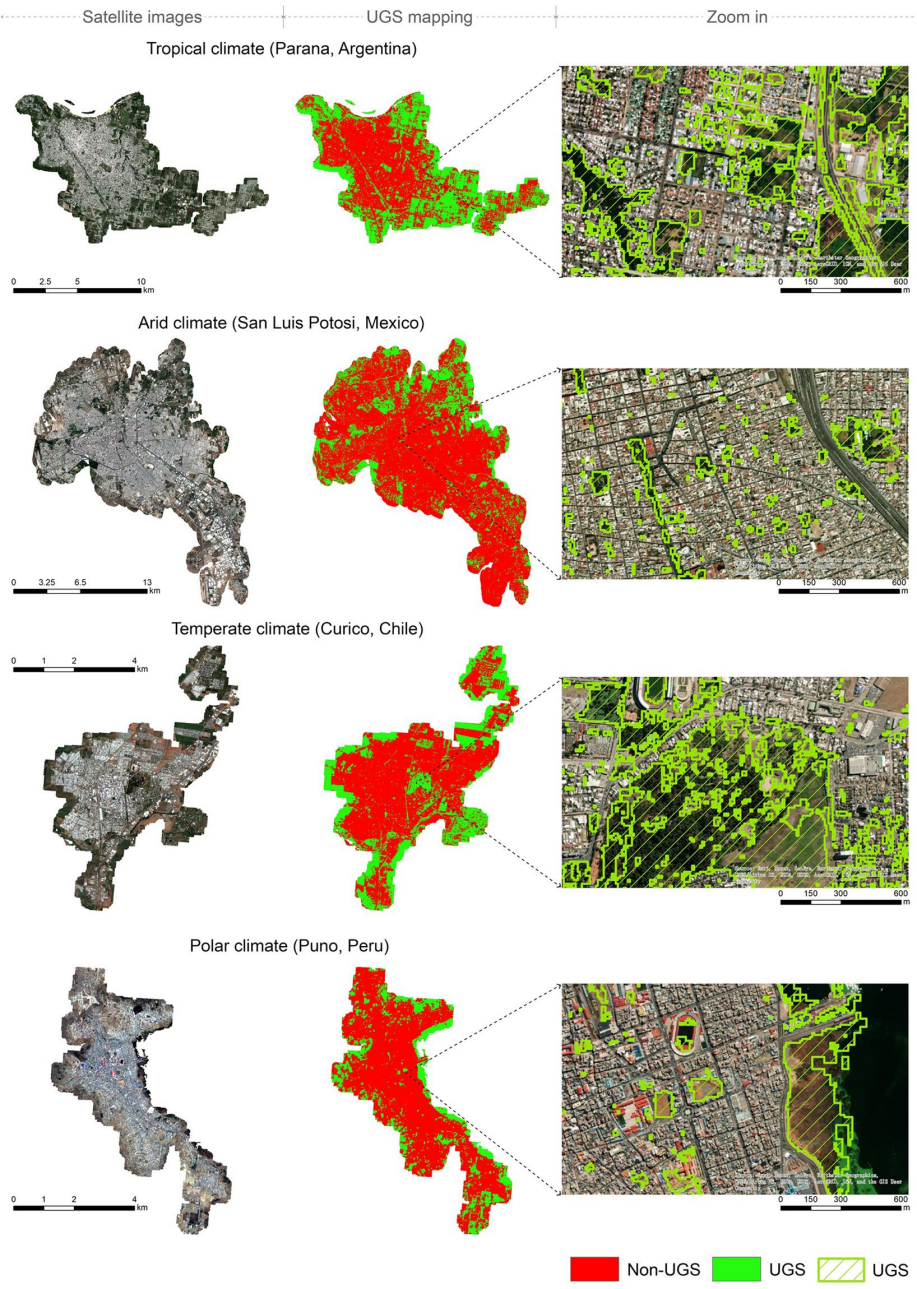
Sample results of the urban green space (UGS) map stratified by climate types. The left panel presents Sentinel-2 satellite images used for UGS mapping, the middle panel presents UGS mapping results, and the right panel zooms to example locations within the city to illustrate the UGS mapped.

The UGS map includes three sets of files: (1) binary UGS maps at 10 m spatial resolution in GEOTIFF format, with each of the 371 cities being an individual map. Mapped value of 1 indicates UGS, 0 indicates non-UGS, and no data (with value of −32768) indicates areas outside the mapped boundary or water bodies; (2) a shapefile of mapped boundaries. The boundary file contains city name, country name and its ISO-2 country code, and an ID field linking each city’s boundary to the corresponding UGS map. (3).prj files containing projection information for the binary UGS maps and boundary shapefile. The binary UGS maps are projected with World Geodetic System (WGS) 84/Pseudo-Mercator projected coordinate system (EPSG: 3857)^39^, and the boundary shapefile is projected with WGS 1984 geographic coordinate system (EPSG: 4326)^40^. We deposited the dataset at the figshare repository (10.6084/m9.figshare.19803790)^41^. UGS maps and the boundary file can be processed with GIS software such as ArcGIS and FRAGSTATS. A sample of the UGS map is in Fig. 3.

## Technical Validation

We validated the UGS maps in two steps. First, we performed an accuracy assessment with independently collected validation samples in 11 cities, selected via a stratified random sampling. These validation samples were independent of the training-validation-testing samples derived from OSM, and the resulting accuracy assessment served as an initial statement of accuracy of the UGS maps. Second, to achieve the best quality possible, two independent inspectors visually assessed classification quality for all 371 cities and identified the ones where mapping was unsatisfactory. We performed further sample collection, quality control, and image classification in these cities until their UGS maps were determined as good quality (Fig. 2e).

### Accuracy assessment in cities from stratified random sampling

We collected 3050 validation samples (ground truth) of UGS and non-UGS, independent of the OSM samples, from 11 cities through a two-step stratified random sampling. First, we stratified the cities by three main climate zones in our study area and by tertiles of their testing accuracies from the earlier step. We selected 11 cities from this process. Second, using the mapping results, we conducted stratified random sampling within the 11 cities to identify locations of validation samples while balancing the sizes of UGS and non-UGS validation samples. We collected a total of 3050 validation samples from the 11 cities, and one author labelled the ground truth of these samples by visually interpreting the input Sentinel-2 images and finer-resolution Google Earth images. We used these validation samples to perform an accuracy assessment with all 11 cities pooled together and individually.

The overall accuracy of UGS maps in the 11 cities was 0.87. We also summarized mapping accuracy of individual cities by their climate zones (Table 3). Cities in tropical climate had better accuracies, as UGS in these cities tends to have greener and denser vegetation therefore showing stronger contrast with non-UGS. Cities in temperate and arid climates had lower accuracies, as UGS in these climates tend to have spectral properties and background surface contributions such that UGS is more easily confused with non-UGS.

**Table 3:** Accuracy of UGS maps of randomly selected cities, summarized by their climate zones.

Climate zone	Average	Min	Max	Number of cities
Tropical	0.92	0.87	0.96	5
Arid	0.83	0.80	0.87	3
Temperate	0.83	0.76	0.89	3

### Visual inspection and quality control

To achieve the best mapping quality possible and to deal with cases where OSM data quality and availability were limited, two of the authors visually inspected the initial UGS maps against the input Sentinel-2 images for all 371 cities and identified cities with unsatisfactory mapping results.

To improve mapping quality in these cities, we went back to the sample collection and filtering process and performed additional quality control to OSM polygons (Fig. 2c). Specifically, we refined boundary and corrected labels for the OSM polygons. In cities with limited OSM coverage, we manually added OSM-like polygons. We then used these updated OSM polygons to perform sample collection and filtering and the subsequent image classification. Finally, we visually inspected the updated mapping results and iteratively performed this quality control on OSM polygons and the subsequent steps until the mapping results were satisfactory.

## Usage Notes

Researchers can use our UGS maps to calculate landscape metrics describing the spatial pattern of UGS at the city scale. Landscape metrics, including the percentage of UGS, patch density, perimeter-area ratio, area weighted distance between patches, describe the prevalence, shape complexity, and connectivity of UGS. These metrics have been used to study the relationship between the spatial configuration of UGS and its ecosystem services^14,15^. Landscape metrics can be calculated by software FRAGSTATS, R package landscapemetrics, and Python package PyLandStats.

The spatially explicit nature of the UGS map also enables integration with other spatially explicit datasets such as locations of survey participants and travel routes to measure detailed exposures to UGS at the individual scale. This integration can be achieved via GIS software and programming packages such as ArcGIS, QGIS, GDAL, and GeoPandas.

## Data Availability

We used Google Earth Engine via Python to query Sentinel-2 images and to extract spectral indices and texture from the images. We performed all other steps, including image downloading, PCA, sample collection and filtering, and image classification in Python. Code is available at https://github.com/yangju-90/urban_greenspace_classification.
